# Methyl Jasmonate Reduces Inflammation and Oxidative Stress in the Brain of Arthritic Rats

**DOI:** 10.3390/antiox8100485

**Published:** 2019-10-15

**Authors:** Heloisa V. Pereira-Maróstica, Lorena S. Castro, Geferson A. Gonçalves, Francielli M.S. Silva, Lívia Bracht, Ciomar A. Bersani-Amado, Rosane M. Peralta, Jurandir F. Comar, Adelar Bracht, Anacharis B. Sá-Nakanishi

**Affiliations:** 1 Department of Biochemistry, University of Maringá, 7020900 Maringá, Brazil; heloisavialle@gmail.com (H.V.P.-M.); losacastro@hotmail.com (L.S.C.); gefersonag2@gmail.com (G.A.G.); lbracht@uem.br (L.B.); rmperalta@uem.br (R.M.P.); jfcomar@uem.br (J.F.C.); abracht@uem.br (A.B.); 2 Department of Pharmacology and Therapeutics, University of Maringá, 87020900 Maringá, Brazilcabamado@uem.br (C.A.B.-A.)

**Keywords:** adjuvant-induced arthritis, rheumatoid arthritis, methyl jasmonate, brain mitochondria, hexokinase, oxidative stress

## Abstract

Methyl jasmonate (MeJA), common in the plant kingdom, is capable of reducing articular and hepatic inflammation and oxidative stress in adjuvant-induced arthritic rats. This study investigated the actions of orally administered MeJA (75–300 mg/kg) on inflammation, oxidative stress and selected enzyme activities in the brain of Holtzman rats with adjuvant-induced arthritis. MeJA prevented the arthritis-induced increased levels of nitrites, nitrates, lipid peroxides, protein carbonyls and reactive oxygen species (ROS). It also prevented the enhanced activities of myeloperoxidase and xanthine oxidase. Conversely, the diminished catalase and superoxide dismutase activities and glutathione (GSH) levels caused by arthritis were totally or partially prevented. Furthermore, MeJA increased the activity of the mitochondrial isocitrate dehydrogenase, which helps to supply NADPH for the mitochondrial glutathione cycle, possibly contributing to the partial recovery of the GSH/oxidized glutathione (GSSG) ratio. These positive actions on the antioxidant defenses may counterbalance the effects of MeJA as enhancer of ROS production in the mitochondrial respiratory chain. A negative effect of MeJA is the detachment of hexokinase from the mitochondria, which can potentially impair glucose phosphorylation and metabolism. In overall terms, however, it can be concluded that MeJA attenuates to a considerable extent the negative effects caused by arthritis in terms of inflammation and oxidative stress.

## 1. Introduction

Methyl jasmonate (MeJA) is a cyclopentanone widely spread over the plant kingdom where it functions as signaling molecule associated with biotic and abiotic stress [1]. It was first isolated from the flowers of jasmine (*Jasminum grandiflorum*), whose infusions have been traditionally used to relieve stress, depression, irritability and memory deficit [2]. MeJA has been reported to present anti-tumor activity without affecting healthy cells [3]. In fact, MeJA was revealed to be cytotoxic against various murine and human cancer cell lines [1,3]. In the cancer cells the compound mainly affects the mitochondria, where it detaches the outer membrane-bound hexokinase, stimulates the production of reactive oxygen species (ROS), depletes ATP and induces apoptosis [4,5].

MeJA shares structural similarity with anti-inflammatory prostaglandins and, therefore, has been lately investigated as a promising anti-inflammatory agent [3,6]. The compound itself and its synthetic derivatives inhibit the synthesis of tumor necrosis factor alpha (TNFα), interleukin 1 beta (IL-1β), interleukin 6 (IL-6), prostaglandin E and nitric oxide (NO) in murine macrophages (RAW264.7) stimulated by lipopolysaccharide (LPS) [6,7,8]. A recent study also showed that orally administered MeJA improves the systemic and articular inflammation in rats with adjuvant-induced arthritis, an experimental model of human severe rheumatoid arthritis [9]. Thus, MeJA emerges also as a promising agent for treatment of rheumatoid arthritis and other systemic inflammatory disorders.

Rheumatoid arthritis is a chronic and autoimmune inflammatory disease that can lead to progressive joint destruction and affects approximately 1.0% of the adult population worldwide [10,11]. An accentuated hyperplasia of the synovial membrane and cartilage and intense production of pro-inflammatory cytokines (TNFα, IL-1β and IL-6) are normally associated with rheumatoid arthritis [12]. The overproduction of reactive species and metalloproteinases are additionally stimulated by cytokines and mediate the tissue injury [13,14]. Rheumatoid arthritis is a systemic disease and in addition to affecting the joints, it evokes inflammatory and oxidative alterations in other organs, such as lungs, liver and heart [12]. In fact, the oxidative stress biomarkers are modified in the serum of patients with rheumatoid arthritis and in the extra-articular tissues of animal models of rheumatoid arthritis [14,15,16,17,18,19]. Metabolic alterations also occur in rheumatoid arthritis, as for example, the condition of muscle wasting known as rheumatoid cachexia [20]. The hepatic metabolism is also significantly modified in rats with adjuvant-induced arthritis, a phenomenon associated with pronounced oxidative stress in the organ [17,21,22,23].

Inflammation in rheumatoid arthritis also affects the brain where it causes fatigue and reduced cognitive function [12]. Cerebral atrophy and other structural modifications have been reported in patients with severe rheumatoid arthritis [24,25]. The levels of ROS, NO, lipoperoxides and protein carbonyl groups are increased in the brain of rats with adjuvant-induced arthritis, particularly in the mitochondria, where the transmembrane potential is also increased [26]. These alterations are accompanied by decreased levels of reduced glutathione (GSH) and diminished activities of antioxidant enzymes. Additionally, the activity of the pro-oxidant enzyme xanthine oxidase (XO) and the pro-inflammatory enzyme iNOS are increased in the brain of arthritic rats [26].

An earlier study has shown that intraperitoneally administered MeJA attenuates the memory dysfunction, decreases the levels of prostaglandin E, TNFα and IL-1 and suppresses the expression of COX-2 and iNOS in the brain of mice with LPS-induced neuroinflammation [27]. In addition, MeJA reverses in mice the memory impairment caused by scopolamine and unpredictable chronic mild stress (UCMS), phenomena that were associated with the improvement in the brain’s levels of oxidative stress biomarkers, specifically lipoperoxides and GSH [28,29]. Considering these actions of MeJA on the cerebral tissue of mice, it is reasonable to hypothesize that MeJA will be capable of attenuating the inflammation and oxidative stress in the brain of arthritic rats. The present work was thus planned to investigate the effects of orally administered MeJA on inflammation and on the oxidative status of the brain of rats using the model of adjuvant-induced arthritis. Because orally administered MeJA stimulated ROS production in isolated hepatic mitochondria [9], this work has also evaluated the production of ROS in isolated brain mitochondria from healthy and arthritic rats as well as the activities of several mitochondrial dehydrogenases. Furthermore, the activity of hexokinase was also evaluated, as the liver of arthritic rats presents a considerably enhanced glucose phosphorylation capacity [30]. The data obtained in the current study should allow to infer about the possible actions of MeJA on the brain of patients with severe rheumatoid arthritis.

## 2. Materials and Methods

### 2.1. Chemicals

Methyl jasmonate, *o*-phthalaldehyde (OPT), 2,4-dinitrophenylhydrazine (DNPH), 1,1′,3,3′-tetraethoxypropane, horseradish peroxidase, nitrate reductase, β-nicotinamide adenine dinucleotide (NADH), 2′-7′-dichlorofluorescein-diacetate (DCFH-DA), oxidized dichlorofluorescein (DCF), reduced glutathione (GSH) and oxidized glutathione (GSSG) were purchased from Sigma Chemical Co (St. Louis, MO, USA). All other chemicals were of analytical grade.

### 2.2. Animals and Induction of Arthritis

Male Holtzman rats weighing approximately 180 g were obtained from the Center of Animal Breeding of the University of Maringá (UEM, Maringá, Brazil) and kept in the Animal Care Unit of our laboratory under standard temperature (24 ± 3 °C) in a 12 h light/dark cycle. The animals were maintained in steel cages (three rats/cage) and fed ad libitum with laboratory diet (Nuvilab^®^, Colombo, Brazil). For arthritis induction, animals were subcutaneously injected in the left hind paw with 0.1 mL (500 µg) of Freund’s adjuvant (heat inactivated *Mycobacterium tuberculosis*, derived from the human strain H37Rv), suspended in Nujol^®^ (Sigma Chemical Co-St. Louis, MO, USA) [31]. Rats of similar weights and age were used as controls. The procedures followed the guidelines of the Brazilian Council for the Control of Animal Experimentation (CONCEA) and were previously approved by the Ethics Committee for Animal Experimentation of the State University of Maringá (Protocol number CEUA 4475171016).

### 2.3. Experimental Design

Forty-two animals were randomly distributed into seven groups: controls (C), which received corn oil; controls (C300) treated with MeJA at the dose of 300 mg/kg; arthritic rats (A), which received corn oil; arthritic rats (A75, A150 and A300) treated with MeJA, respectively, at the doses of 75, 150 and 300 mg/kg; and arthritic rats (A_IBU_) treated with ibuprofen (30 mg/kg). This procedure was repeated three times (42 × 3 = 126 rats in total) throughout the study. Animals were orally treated (by gavage) once a day with MeJA, corn oil or ibuprofen during the 18 days after arthritis induction. The doses of MeJA were defined according to previous studies showing that they are biologically active and well tolerated when the compound is administered to rodents [9,32].

### 2.4. Brain Homogenate Preparation and Mitochondria Isolation

Fasted (12 h) rats were anesthetized with an overdose of sodium thiopental (100 mg/kg) and the brain immediately removed, freeze-clamped and stored in liquid nitrogen. The tissue was homogenized in a van Potter–Elvehjem homogenizer with nine volumes of ice-cold 0.1 M potassium phosphate buffer (pH 7.4) and an aliquot was separated as total homogenate. The remaining homogenate was centrifuged (11,000*g*/15 min) and the supernatant separated as homogenate supernatant. Brain mitochondria were isolated by differential centrifugation as previously described [33] with some modifications. The fresh brain was placed in ice-cold solution containing 200 mM mannitol, 76 mM sucrose, 1 mM ethylene glycol-bis(2-aminoethylether)-N,N,N′,N′-tetraacetic acid (EGTA), 0.1 mM PMSF (phenylmethylsulfonyl fluoride) and 10 mM 2-amino-2-hydroxymethyl- propane-1,3-diol (TRIS; pH 7.4). The brain was cut into small pieces and homogenized in a van Potter–Elvehjem homogenizer. The homogenate was then first centrifuged at 650*g* for 10 min and the mitochondria in the supernatant were sedimented by centrifuging at 8000*g* (10 min). The resulting mitochondria were washed and resuspended in the same buffer. An aliquot of this preparation was used to prepare disrupted mitochondria by a repeated freeze-thawing procedure in liquid nitrogen.

### 2.5. Brain Oxidative Stress and Inflammatory Parameters

Protein carbonyl groups were measured spectrophotometrically in the brain homogenate supernatant using 2,4-dinitrophenylhydrazine (DNPH) as previously described [34]. The molar extinction coefficient (ε) of 2.20 × 10^4^ M^−1^ cm^−1^ was used in the calculations.

The levels of lipoperoxides were quantified by means of the thiobarbituric acid reactive substances (TBARS) assay [35]. The content in TBARS was calculated from the standard curve prepared with 1,1′,3,3′-tetraethoxypropane.

The content of ROS was quantified via the 2′-7′-dichlorofluorescein-diacetate (DCFH-DA) assay [36], which quantifies the oxidation of DCFH to the fluorescent 2′,7′-dichlorofluorescein (DCF) in the presence of ROS. The formation of DCF was measured by spectrofluorimetry (Shimadzu RF-5301; 504 nm for excitation and 529 nm for emission). Although ROS have short half-lives, decomposition in frozen tissue is not very pronounced and the fractional loss and inactivations are independent of their concentrations, so that the proportions between the various conditions are preserved when they are assayed at the same time [9,17].

The rate of mitochondrial ROS production was estimated by measuring the linear fluorescence increase (504 nm for excitation and 529 nm for emission) due to DCF formation from DCFH via oxidation by H_2_O_2_ in the presence of horseradish peroxidase [37]. Briefly, intact mitochondria (0.5 mg) were suspended in 2 mL of a mixture containing 250 mM mannitol, 1.36 μM DCFA-DA, 10 mM HEPES buffer (pH 7.2), 10 μM rotenone and 10 mM succinate as respiratory substrate. Fluorescence was recorded during 10 min under agitation. The results were expressed as nmol min^−1^ (mg protein)^−1^.

The production of NO was indirectly quantified by measuring the levels of nitrite plus nitrate in the brain homogenate supernatant. Nitrate was first converted into nitrite by adding nitrate reductase and the total nitrite was quantified by the Griess reagent [38].

Reduced (GSH) and oxidized (GSSG) glutathione were measured in the brain homogenate by spectrofluorimetry (excitation at 350 nm and emission at 420 nm) with the *o*-phthalaldehyde (OPT) assay [39]. The activities of catalase, superoxide dismutase (SOD), xanthine oxidase (XO) and myeloperoxidase (MPO) were assayed by spectrophotometry in the supernatant of the brain homogenate. The catalase activity was estimated spectrophotometrically at 240 nm using H_2_O_2_ as substrate [40]. The activity of SOD was estimated by the pyrogallol autoxidation method [41]. The activity (MPO) was measured by spectrophotometry with *o*-dianisidine [42]. The XO activity was estimated as the increase in absorbance at 295 nm due to uric acid formation [43].

### 2.6. Mitochondrial Transmembrane Potential (ΔΨ_m_) and Enzyme Activities

Mitochondrial membrane energization (transmembrane potential; ΔΨ_m_) was measured by spectrofluorimetry using safranin as fluorescent probe [44]. The wavelengths for excitation and emission were 520 and 580 nm, respectively. Energization was achieved by addition of 50 μM succinate plus 2 μM rotenone and the full de-energization was achieved by the addition of carbonyl cyanide-4-(trifluoro-methoxy) phenylhydrazone (FCCP; 10 μM).

The activity of NADH dehydrogenase in disrupted mitochondria was estimated by spectrophotometry at 420 nm using potassium ferricyanide as the electron acceptor and the results were calculated using the molar extinction coefficient (ε) of 1.04 × 10^3^ M^−1^ cm^−1^ [45].

The ATPase activity was measured in intact (coupled and uncoupled) and disrupted mitochondria as previously described [46]. Briefly, intact mitochondria were incubated in a medium (0.5 mL) containing 50 mM KCl, 0.2 M sucrose, and 10 mM Tris–HCl (pH 7.4) plus 0.2 mM EGTA and 5.0 mM ATP for 20 min, at 37 °C. The reaction was initiated by the addition of ATP and stopped with 5% trichloroacetic acid. The ATPase activity was quantified by measuring inorganic phosphate release from ATP.

The activity of succinate dehydrogenase was measured by spectrophotometry (500 nm) in a medium containing 100 mM triethanolamine (pH 8.3), 0.5 mM EDTA, 2 mM KCN, 6.5 μM phenazine methosulfate, 0.6 mM iodonitrotetrazolium, and aliquots of disrupted mitochondria [40]. The reaction was initiated with the addition of 10 mM succinate. Values were calculated using the molar extinction coefficient (ε) of reduced iodonitrotetrazolium (1.93 × 10^4^ M^−1^ cm^−1^).

The activity of malate dehydrogenase was assayed by spectrophotometry at 340 nm in a medium (1.5 mL) containing 120 mM phosphate buffer (pH 7.8), 0.25 mM NADH, and aliquots of the supernatant obtained after centrifuging disrupted mitochondria at 10,000*g*. The reaction was started by the addition of 0.1 mM oxaloacetate [47]. Values were calculated using the molar extinction coefficient (ε) of NADH (6.22 × 10^3^ M^−1^ cm^−1^).

The activity of the NADP^+^-dependent isocitrate dehydrogenase was assayed in a reaction medium (1 mL) containing 0.1 M TRIS (pH 7.4), 2 mM MgCl_2_, 2 mM NADP^+^, and aliquots of the supernatant [48]. The reaction was started by the addition of 1.25 mM isocitrate and the increase in absorbance was monitored at 340 nm (ε = 6.22 × 10^3^ M^−1^ cm^−1^). 

The activity of L-glutamate dehydrogenase was measured in a reaction medium (1 mL) containing 50 mM triethanolamine (pH 8.0), 0.1 M ammonium sulfate, 95 μM NADH, 2.5 mM EDTA, 1 mM ADP, and aliquots of the supernatant described above [49]. The reaction was started by addition of α-ketoglutarate (8.0 mM) and the decrease in absorbance was monitored at 340 nm.

The activity of α-ketoglutarate dehydrogenase was measured in a medium containing 100 mM phosphate buffer (pH 7.4), 2  mM NAD^+^, 0.2 mM thiamine pyrophosphate, 1 mM MgCl_2_, 0.1% Triton X-100, 0.3 mM dithiothreitol, 10 mM α-ketoglutarate and aliquots of disrupted mitochondria suspensions [40]. The reaction was initiated by the addition of coenzyme A (0.2 mM) and monitored spectrophotometrically as the reduction of NAD^+^ at 340 nm (ε = 6.22 × 10^3^ M^−1^ cm^−1^). 

### 2.7. Brain Hexokinase (HK) Activity

Fresh brains were homogenized in medium containing 200 mM mannitol, 76 mM sucrose, 1 mM EGTA, 0.1 mM PMSF and 10 mM TRIS (pH 7.4). The homogenate was centrifuged at 650*g* for 8 min. The activity of the hexokinase (HK) in the cytosol was measured using the liquid fraction obtained by an additional centrifugation of the brain homogenate supernatant (40 min at 29,000*g*) [50]. This procedure precipitates the mitochondria and other cell components. The incubation medium contained in a final volume of 1 mL: 100 mM Tris-HCl (pH 7.2), 20 mM glucose, 2 mM ATP, 10 mM MgCl_2_, 1.5 mM NAD⁺, 5 units of glucose 6-phosphate dehydrogenase from *Leuconostoc mesenteroides* and 50 µL of high speed centrifugation supernatant. The reaction was monitored spectrophotometrically at 340 nm (ε = 6.22 × 10^3^ M^−1^ cm^−1^).

### 2.8. Statistical Analysis

Statistical analysis was done using the GraphPad Prism Software (version 5.0). Statistical significance was inferred from ANOVA one-way with Newman–Keuls post-hoc testing. The 5% level of significance was adopted (*p* < 0.05).

## 3. Results

### 3.1. Indicators of Inflammation and Oxidative Stress

**Figure 1:**
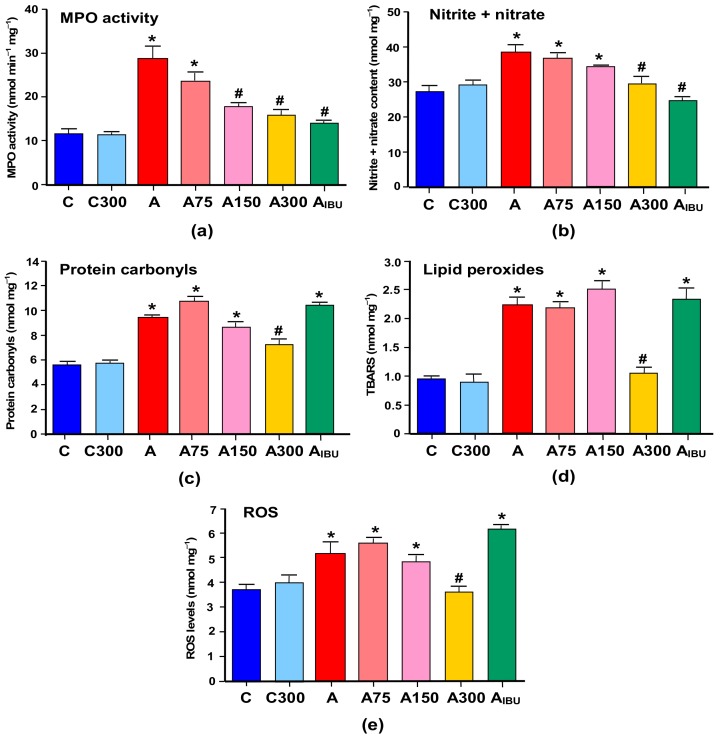
Effects of the methyl jasmonate (MeJA) treatment on parameters of inflammation and oxidative stress in the brain. The activity of (**a**) myeloperoxidase (MPO); and (**b**) the levels of nitrite plus nitrate; (**c**) protein carbonyl groups; (**d**) thiobarbituric acid reactive substances (TBARS); and (**e**) reactive oxygen species (ROS) were measured as described in Materials and Methods. C, controls treated with corn oil; C300, controls treated with 300 mg/kg MeJA; A, arthritic rats treated with corn oil; A75, A150 and A300, arthritic rats treated with 75, 150 and 300 mg/kg MeJA, respectively; A_IBU_, arthritic rats treated with 30 mg/kg ibuprofen. Data are the means ± standard errors of the mean of five animals for each experimental condition. Statistical analysis: ANOVA one-way with Newman–Keuls post-hoc testing. * *p* < 0.05, different from the controls (C); #*p* < 0.05, different from non-treated arthritic rats (A).

The results of the measurements of indicators of inflammation and oxidative stress in the brain of the various groups of experimental animals are shown in Figure 1. Figure 1a,b illustrates the effects of arthritis and the MeJA and ibuprofen treatments on the myeloperoxidase (MPO) activity and the nitrite plus nitrate levels. The latter are indicators for the NO production. The MeJA treatment did not affect the MPO activity in healthy rats. In arthritic rats, however, in which the activity of MPO was increased 2.5-fold, the MeJA treatment caused progressive diminutions as the doses were increased. At the highest dose (300 mg/kg) the MPO activity was close to the activity in the control animals and in the rats treated with ibuprofen. The levels of nitrite plus nitrate were increased approximately 1.4-fold in the brain of arthritic rats. Here again the MeJA treatment did not modify these levels in control rats. In arthritic rats both the MeJA and the ibuprofen treatments diminished the elevated nitrite plus nitrate levels. For the MeJA dose of 300 mg/kg the nitrite plus nitrate levels were very close to those observed in control rats or in ibuprofen-treated rats.

Figure 1c–e shows the levels of protein carbonyls, lipid peroxides (TBARS) and reactive oxygen species (ROS) that were found in the brain of arthritic and control rats and the effects of MeJA and ibuprofen treatments. The levels of carbonyl groups and TBARS were increased 1.7- and 2.2-fold, respectively, in the brain of arthritic rats. No modifications were found when MeJA was given to healthy rats. In arthritic rats the MeJA doses of 75 and 150 mg/kg were also ineffective in modifying the protein carbonyls and TBARS levels. Actually, there was even a small tendency toward higher values, though lacking statistical significance. Treatment with 300 mg/kg MeJA, however, produced clear decreases in the levels of carbonyl groups and TBARS to values close to the control ones. The levels of ROS were increased 1.4-fold in the brain of arthritic rats. The MeJA treatment of control rats was without effect. Here again the lower MeJA doses were ineffective and only the 300 mg/kg dose produced a significant and pronounced decrease to a value close to that of the control condition. Treatment of arthritic animals with ibuprofen did not improve any of the oxidative stress indicators.

### 3.2. GSH Levels

**Figure 2:**
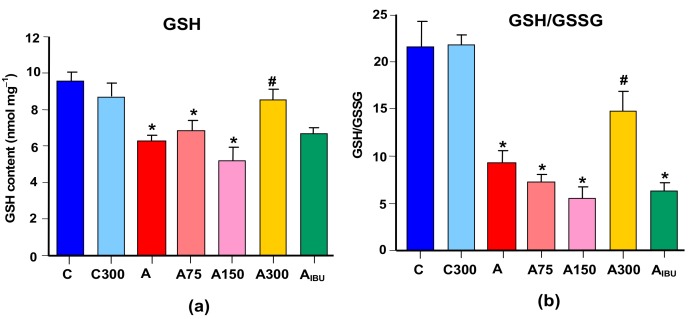
Effects of the MeJA treatment on (**a**) glutathione (GSH) levels and (**b**) GSH/ oxidized glutathione (GSSG) ratios. Symbols are those defined in Figure 1. Data are the means ± standard errors of the means of five animals for each experimental condition. Statistical analysis: ANOVA one-way with Newman–Keuls post-hoc testing. *p < 0.05, different from the controls (C); #*p* < 0.05, different from non-treated arthritic rats (A).

Figure 2 shows the levels of reduced glutathione (GSH) and the GSH/GSSG ratio. The levels of GSH and the GSH/GSSG ratio were 35% and 70% lower, respectively, in the brain of arthritic animals. The level of GSSG was not modified by arthritis, but it was slightly increased in rats treated with 75 and 150 mg/kg MeJA and ibuprofen (not shown). Treatment of arthritic rats with 300 mg/kg MeJA, but not with ibuprofen, increased the level of GSH to a value close to the control one. In consequence, an almost similar increase in the GSH/GSSG ratio was also found at the 300 mg/kg dose, although a decreasing tendency was apparent at lower doses.

### 3.3. Mitochondrial ROS Generation and Membrane Potential

**Figure 3:**
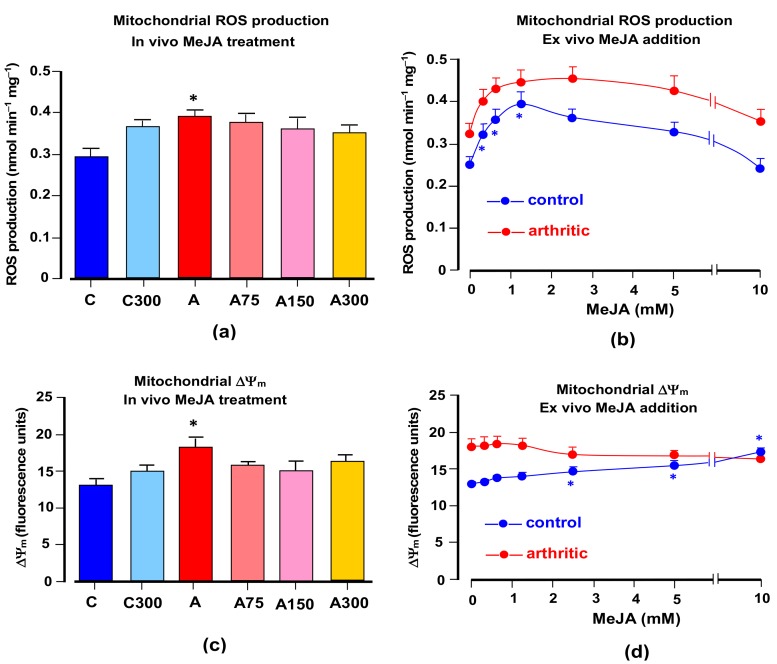
Effects of MeJA on ROS production and membrane potential in brain mitochondria from healthy and arthritic rats. (**a**) ROS production in mitochondria isolated from healthy and arthritic rats, treated or not with different MeJA doses (C, controls; C300, controls treated with 300 mg/kg MeJA; A, arthritic rats; A75, A150 and A300, arthritic rats treated with 75, 150 and 300 mg/kg MeJA, respectively). (**b**) ROS production by brain mitochondria isolated from healthy and arthritic rats and incubated with varying concentrations of MeJA. (**c**) Membrane potential (ΔΨ_m_) of brain mitochondria isolated from healthy and arthritic rats, treated or not with different MeJA doses. (**d**) Membrane potential (ΔΨ_m_) of brain mitochondria isolated from healthy and arthritic rats and incubated with varying concentrations of MeJA. Data are the means ± standard errors of the mean of five animals for each experimental condition. Statistical analysis: ANOVA one-way with Newman–Keuls post-hoc testing. *p < 0.05, different from the corresponding controls.

In isolated respiring rat liver mitochondria, it has been found that MeJA increases net ROS generation [9]. For this reason, experiments were done in which the action of MeJA treatment on ROS generation by brain mitochondria was measured. Figure 3a shows the results obtained when brain mitochondria isolated from rats under various conditions were incubated in a medium containing succinate as the electron donor. ROS generation was measured as the fluorescence increase due to the formation of the indicator DCF. In mitochondria from arthritic rats, ROS generation was clearly increased by a factor of 1.36. Treatment with MeJA did not reverse this modification although there was some tendency in this direction. There was also a small tendency toward increasing ROS generation in mitochondria from healthy rats treated with MeJA. ROS generation is a dynamic variable which could be dependent on the continuous presence of MeJA. Since the latter is no longer present in mitochondria from treated rats, attempts were made at investigating the short term effects of the compound. The results shown in Figure 3a represent the possible reversible and short-term effects of MeJA on ROS generation in isolated mitochondria from healthy and arthritic rats in the range of up to 10 mM. They show that the compound increased ROS generation in brain mitochondria from both healthy and arthritic rats. The increments relative to the starting conditions were similar for both healthy and arthritic conditions in the range up to 1.25 mM. After this concentration there was a declining tendency, more pronounced in mitochondria from healthy rats.

It is generally accepted that ROS generation in mitochondria shows a positive correlation with the membrane potential [51]. The measurements illustrated by Figure 3c,d were done in order the explore this relationship in terms of the effects of arthritis and MeJa. As described in the Materials and Methods section, energization was achieved by the addition of succinate, the same substrate used in the experiments in which ROS generation was measured. Figure 3c reveals a clearly increased membrane energization in mitochondria from arthritic rats. This correlates nicely with the increased rates of ROS generation shown in Figure 3a. The effects of MeJA treatment, however, are unclear, as no statistically significant modifications were detected even though there is a general tendency toward a lower energization. When mitochondria isolated from control and arthritic rats were incubated with varying MeJA concentrations in the range of up to 10 mM in order to explore possible reversible actions (Figure 3d), only minimal modifications were observed. These were restricted to the control rats, in which there was a small increment at high MeJA concentrations.

### 3.4. Antioxidant and Prooxidant Enzyme Activities

**Figure 4:**
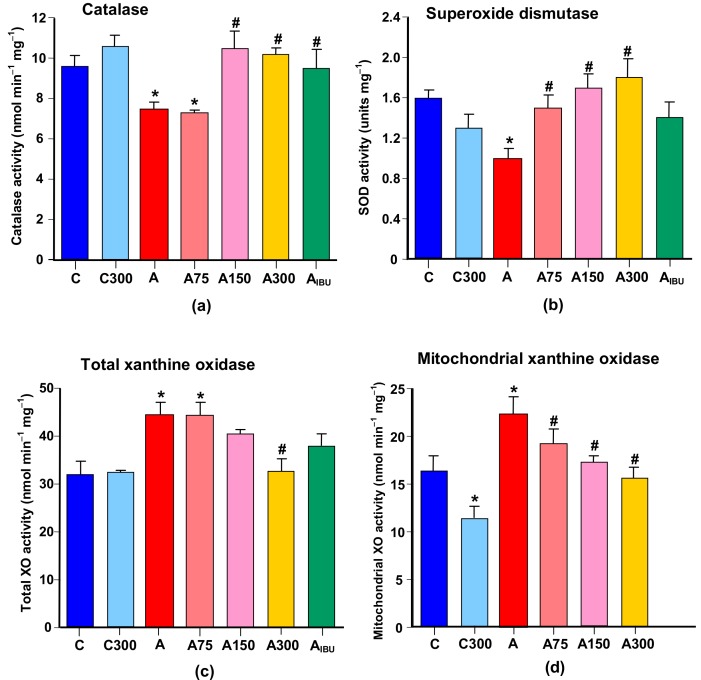
Effects of MeJA treatment on enzymes linked to the oxidative homeostasis. (**a**) Catalase; (**b**) superoxide dismutase; (**c**) total xanthine oxidase; (**d**) mitochondrial xanthine oxidase. Abbreviations: C, controls; C300, controls treated with 300 mg/kg MeJA; A, arthritic rats; A75, A150 and A300, arthritic rats treated with 75, 150 and 300 mg/kg MeJA, respectively; A_IBU_, arthritic rats treated with 30 mg/kg ibuprofen. Data are the means ± standard errors of the mean of five animals for each experimental condition. Statistical analysis: ANOVA one-way with Newman–Keuls post-hoc testing. *p < 0.05, different from the controls (C); # *p* < 0.05, different from non-treated arthritic rats (A).

Figure 4a,b shows the influence of arthritis and the subsequent MeJA treatment on the activities of the antioxidant enzymes catalase (CAT) and superoxide dismutase (SOD). The CAT and SOD activities were 25% and 40% lower, respectively, in the brain of arthritic rats. Treatment with 150 and 300 mg/kg MeJA or ibuprofen increased the catalase activity to values close to the control ones. Treatment of arthritic rats with 75, 150 and 300 mg/kg MeJA, but not with ibuprofen, increased the SOD activity to values close to the control ones.

Figure 4c,d illustrates the actions of arthritis and MeJA on the activity of xanthine oxidase (XO), which can be considered a prooxidant enzyme whose activity is usually recognized as an important source of reactive oxygen species in the brain [26]. In the brain homogenate from arthritic rats the activity of XO was increased 1.4-fold. The treatment with 300 mg/kg MeJA almost abolished this increase. The activity of XO was also increased 1.4-fold in isolated brain mitochondria of arthritic rats. In this case the treatment of arthritic rats with MeJA showed a well-defined dose-dependent diminution to values close to the control ones.

### 3.5. Mitochondrial Enzymes Linked to Oxidative Metabolism

**Figure 5:**
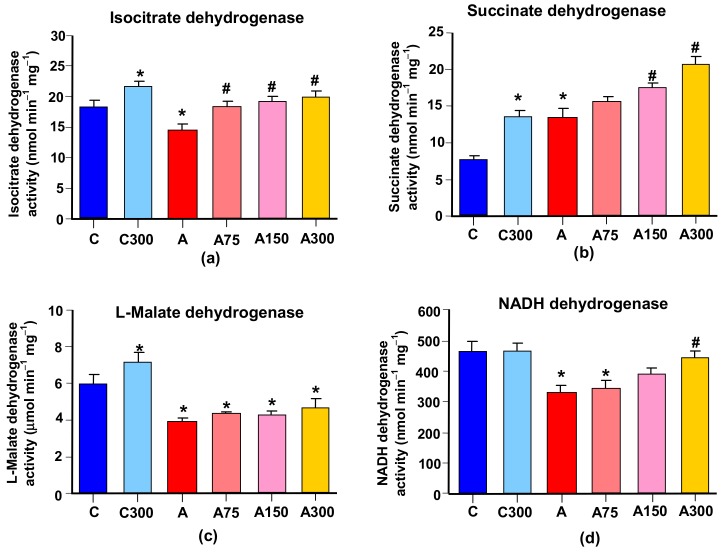
Effects of MeJA treatment on selected mitochondrial enzyme activities. (**a**) Succinate dehydrogenase; (**b**) isocitrate dehydrogenase; (**c**) L-malate dehydrogenase; (**d**) NADH dehydrogenase. Abbreviations: C, controls; C300, controls treated with 300 mg/kg MeJA; A, arthritic rats; A75, A150 and A300, arthritic rats treated with 75, 150 and 300 mg/kg MeJA, respectively; A_IBU_, arthritic rats treated with 30 mg/kg ibuprofen. Data are the means ± standard errors of the mean of five animals for each experimental condition. Statistical analysis: ANOVA one-way with Newman–Keuls post-hoc testing. * *p* < 0.05, different from the controls (C); # *p* < 0.05, different from non-treated arthritic rats (A).

Several mitochondrial enzymes linked to oxidative metabolism were assayed with the purpose of searching for possible modifications in energy metabolism and in reactions leading to the production of NADPH for feeding the glutathione cycle with reducing equivalents. To the latter category belong L-glutamate dehydrogenase (which also operates with NADP^+^) and the NADP^+^-dependent isocitrate dehydrogenase. The activity of the first one was not modified by arthritis or by the subsequent MeJA treatment (not shown). The NADP^+^-dependent isocitrate dehydrogenase, however, was diminished in arthritic rats by 23%, as shown in Figure 5a. The MeJA treatment increased this enzyme in both healthy and arthritic rats. In the latter, this effect led to a complete prevention of the diminution caused by arthritis.

The other enzymatic activities linked to oxidative metabolism that were measured were ATPase, α-ketoglutarate dehydrogenase, L-malate dehydrogenase, succinate dehydrogenase and NADH- dehydrogenase. No modifications by arthritis or MeJA treatment were found for the first two. The other three, however, suffered modifications. Succinate dehydrogenase was increased by a factor of 1.8 in arthritic rats; almost the same increment was caused by the MeJA treatment of healthy rats. The latter effect was apparently maintained in arthritic rats in which the already higher activity was further increased by the MeJA treatment. For the 300 mg/kg dose this led to an increase of 2.76-fold when compared to the healthy controls. The activities of the other two enzymes were diminished by arthritis. The L-malate dehydrogenase activity was diminished by 30%. Although the MeJA treatment of healthy rats produced an increase in the L-malate dehydrogenase activity, this effect was not prominent enough in arthritic rats so as to conduct to a significant recovery. The diminution of NADH dehydrogenase caused by arthritis amounted to 31%. The treatment of healthy rats with 300 mg/kg MeJA did not produce modifications. However, the same treatment of arthritic rats almost completely prevented the diminution of the NADH-dehydrogenase activity.

### 3.6. Hexokinase Activity

**Figure 6:**
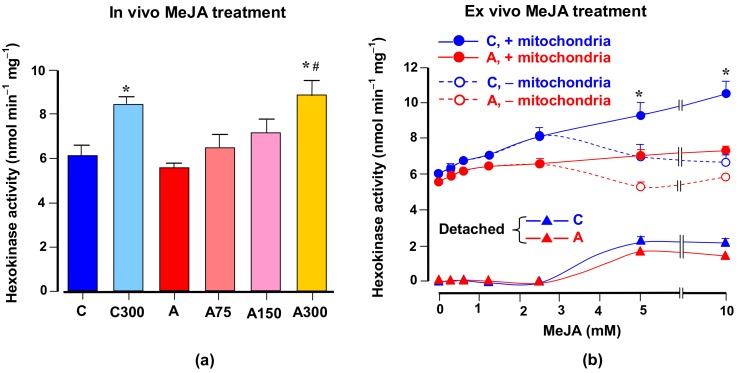
Effects of MeJA treatment on hexokinase activity. (**a**) Hexokinase activities in the cytosolic fraction of brains from healthy and arthritic rats, treated or not with different MeJA doses (C, controls; C300, controls treated with 300 mg/kg MeJA; A, arthritic rats; A75, A150 and A300, arthritic rats treated with 75, 150 and 300 mg/kg MeJA, respectively). (**b**) Hexokinase activities in the cytosolic fraction of brains from healthy and arthritic rats incubated with various MeJA concentrations with or without previous centrifugation to eliminate mitochondria and other cell components. Data are the means ± standard errors of the mean of five animals for each experimental condition. Statistical analysis: ANOVA one-way with Newman–Keuls post-hoc testing; *p < 0.05, different from the corresponding controls (C); #*p* < 0.05, different from non-treated arthritic rats (A).

The liver of arthritic rats presents a considerably increased glucose phosphorylation capacity [30], which is diminished when the rats are treated with MeJA [9]. For these reasons and because glucose oxidation may be related to the oxidative state of the brain tissue, experiments were done in which the influence of arthritis and MeJA on the hexokinase activity was investigated. Figure 6 illustrates the results that were obtained. Figure 6a shows the measurements of the hexokinase activity in the cytosolic fraction of brains from healthy and arthritic rats, treated or not with the current different MeJA doses. Arthritis had no significant effect on the hexokinase activity present in the cytosolic fraction. The MeJA treatment, on the other hand, increased the hexokinase activity in the cytosolic fraction obtained from healthy rats. A similar effect was found in arthritic rats treated with MeJA, with a maximal effect at the dose of 300 mg/kg.

In brain cells the hexokinase is attached to the mitochondrial membrane and it is known that MeJA is able to detach the enzyme [5]. Since mitochondria were absent in the cytosolic fraction used for the hexokinase assay, the possibility exists that the higher hexokinase activities found in the brain of MeJA-treated animals can actually represent detached enzyme due to the continuous presence of MeJA in the tissue. To investigate this possibility the series of experiments shown in Figure 6b were done. MeJA was added at varying concentrations to the low-speed centrifugation supernatant of the brain homogenate either before or after the high-speed centrifugation for precipitating the mitochondria and other cell components. The time of exposure to MeJA was in all cases 30 min. The hexokinase activity in the cytosolic fractions was represented against the MeJA concentrations. Up to the MeJA concentration of 2.5 mM, precipitation of the mitochondria did not modify the activity of hexokinase found in the cytosolic fraction, although a small increasing tendency was found for both types of incubations. Above this concentration, however, the hexokinase activity continued to increase with the MeJA concentration in those preparations in which the mitochondria were still present when the compound was added. In the preparations in which the mitochondria had been precipitated previous to the addition of MeJA, the hexokinase activity ceased to increase. The enhancement of the activity when the incubation containing mitochondria was used probably reflects the detachment of the hexokinase bound to the organelles caused by MeJA. The detached hexokinase, shown in Figure 6b, calculated as the difference between incubations with and without mitochondria, was similar for the healthy and arthritic condition.

## 4. Discussion

**Figure 7:**
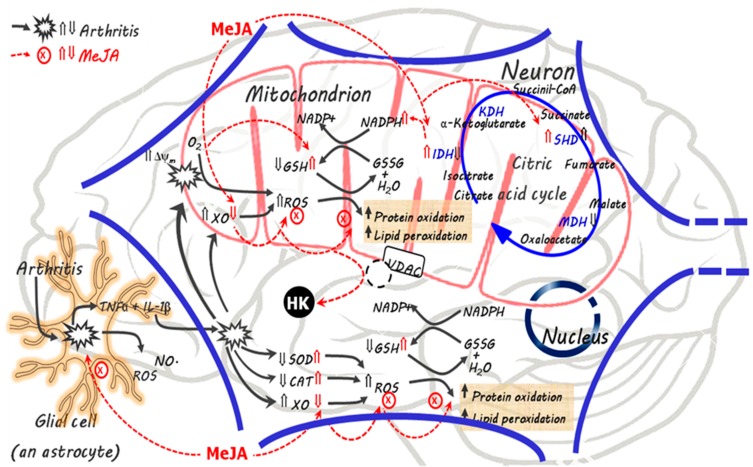
Events modifying inflammation and the oxidative state in the brain of rats with adjuvant-induced arthritis and the actions of methyl jasmonate (MeJA). The scheme is based mainly on the results of the present study and literature data. The symbol ⇑ means up-regulation and ⇓ means down-regulation. Black arrows indicate events in the absence of MeJA and red arrows indicate the effects of MeJA. Abbreviations: TNF-α, tumor necrosis factor alpha; IL-1β, interleukin 1 β; GSH, reduced glutathione; GSSG, oxidized glutathione; ROS, reactive oxygen species; NO, nitric oxide; HK, hexokinase; SOD, superoxide dismutase; CAT, catalase; IDH, isocitrate dehydrogenase; MDH, malate dehydrogenase; KDH, alpha-ketoglutarate dehydrogenase; SDH, succinate dehydrogenase; XO, xanthine oxidase; Δψ_m_, mitochondrial membrane potential; VDAC: voltage-dependent anion channel.

The actions of MeJA in the brain of arthritic rats clearly point in the direction of a diminution of both inflammation and oxidative stress. The modifications that MeJA caused, however, were multivariate and complex, and frequently not restricted to the arthritic rat. The scheme in Figure 7 offers an overview of the actions of MeJA in the brain cells in opposition to the modifications induced by arthritis, which should be a helpful guide in the discussion that follows. The scheme in Figure 7 assumes that MeJA easily crosses biological membranes including the blood brain barrier, as demonstrated experimentally and indeed expected from a lipid soluble substance [3,52]. The black arrows refer mainly to the modifications caused by arthritis whereas the red dotted arrows indicate the main sites of action of MeJA. In the brain, the increased oxidative stress induced by arthritis has been attributed to both an increased production of ROS and an impaired ROS scavenging system [26]. In fact, previous reports and the results of this study show that the activity of XO and mitochondrial ROS production are increased in the arthritic condition, while the GSH levels and the activity of superoxide dismutase and catalase are reduced in the brain [26,53]. This imbalance between pro- and antioxidant systems results in higher levels of ROS, protein oxidation and lipoperoxides in the arthritic brain.

In the present study the indicators of inflammation in the brain of arthritic rats that were measured are the activity of MPO and the level of nitrite plus nitrate as an indicator of the NO concentration. The MPO activity has been considered as one of the best inflammatory and oxidative stress markers for several inflammatory diseases [54]. The nitrite (NO_2_^−^) plus nitrate (NO_3_^−^) levels, on the other hand, are an index of NO production and indirectly also of the inflammatory cytokines. Inflammatory cytokines stimulate glial cells to produce nitric oxide (NO) by the inducible NO synthase (iNOS) in the brain [55,56]. Both the activity of MPO and the nitrite plus nitrate levels, appeared substantially increased in arthritic rats and both were reduced to the control levels by the MeJA treatment at the doses of 150 and 300 mg/kg. These effects were similar to ibuprofen (30 mg/kg), an anti-inflammatory agent currently used to attenuate the symptoms of rheumatoid arthritis. This is consistent with a previous study showing that orally administered MeJA improves the systemic and articular inflammation in rats with adjuvant-induced arthritis [9]. The mechanism by which MeJA decreases inflammation is not yet sufficiently known, but it has been reported that it influences the activation of nuclear factor kappa B (NF-𝜅B). Experiments have shown that MeJA attenuates the LPS-induced activation of NF-𝜅B in RAW267.4 cells and reduces the expression of NF-𝜅B in the brain of mice with LPS-induced neuroinflammation [7,27]. In the latter, intraperitoneally administered MeJA additionally inhibits the production of IL-1β, IL-6, TNFα and prostaglandin E, possibly by glial cells, as illustrated by Figure 7. It also downregulates the expression of iNOS and COX-2 in the brain. These results were recently corroborated by the observation that MeJA reduces the levels of TNF-α and IL-6 and nitrite in the brain of mice with neuroinflammation induced by rotenone, a phenomenon that is also likely to be related to the downregulation of the NF-κB expression [57]. In addition, in mice, MeJA improves the memory dysfunction associated with the neuroinflammation [27]. The effective doses of MeJA in the present study, i.e., 150 and 300 mg/kg, may be relatively high for clinical purposes, but it must be stressed that they have been associated to a notable absence of toxicity [3,9,32].

Comparison of the dose dependences of the decreases in the inflammatory indicators (MPO and nitrite plus nitrate), on one side, with the dose dependences of the decreases in the oxidative stress indicators (protein carbonyls, TBARS and ROS), on the other side, reveals that there was a dose shift toward higher ones in the latter actions (see Figure 1). Diminution of oxidative stress occurred only at the highest dose. At the lowest doses there was even an increasing tendency of the oxidative stress indicators. This may bear relation to the fact that MeJA actually exerts a dual role, i.e., although it certainly acts as an antioxidant agent, it can also be regarded as a prooxidant agent in spite of its anti-inflammatory action. The prooxidant action is exerted in the respiratory chain. The latter is an important source of reactive oxygen species [58] and our experimental data show that the compound increases ROS output in respiring brain mitochondria in accordance to previous observations in rat liver mitochondria [9]. This effect depends on the presence of MeJA as it was not preserved when the mitochondria were isolated from the brain tissue of treated rats. Furthermore, this action was not different in healthy and arthritic rats. It is unlikely that the phenomenon bears relation to the membrane potential, as MeJA did not modify this parameter. Its mechanism remains to be clarified.

The net diminution in oxidative stress caused by MeJA derives, thus, from its effects on ROS generation systems other than the mitochondrial electron transport chain or from its role as an enhancer of the antioxidant defenses. In the first category one may include ROS-producing enzymes whose activity is increased by the inflammatory mediators of arthritis. One such enzyme, which is generally considered an important source of ROS in the brain, namely xanthine oxidase, was analyzed in the present work (see Figure 7). Xanthine oxidase, under specific circumstances, is able to generate H_2_O_2_, superoxide anion and nitric oxide [26,59,60]. Its activity, which was substantially increased by arthritis, was gradatively diminished to control values by MeJA, especially in the mitochondria. This may be an important factor that helps to reduce the production of ROS in the mitochondria under in vivo conditions. With respect to the antioxidant defenses, MeJA clearly restored the activity of both catalase and superoxide dismutase. These actions can be attributed at least in part to an inhibition of the production of proinflammatory cytokines (see Figure 7). MeJA also caused a recovery in the GSH levels and in the GSH/GSSG ratio, corroborating the conclusion that the compound enhances the antioxidant defenses. In this respect the increase in the activity of isocitrate dehydrogenase induced by MeJA may play a significant role as the production of NADPH can occur directly via the action of this enzyme. Consequently, preservation of the activity of the mitochondrial isocitrate dehydrogenase by the MeJA treatment in arthritic rats is expected to contribute to the preservation of a high NADPH/NADP^+^ ratio. This, in turn, will contribute to an increased regeneration of GSH and reduction of the oxidative stress via the glutathione system. Similar MeJA actions were also found in the liver of arthritic rats [9], which were attributed to microRNA 101-mediated upregulation of the nuclear factor erythroid 2-related 2 (Nfr2), a redox-sensible transcription factor that upregulates antioxidant defense genes, including catalase and enzymes required for GSH synthesis and sources of NADPH like isocitrate dehydrogenase [61,62,63]. 

In addition to isocitrate dehydrogenase, adjuvant-induced arthritis also modified the activities of other respiratory enzymes, namely NADH dehydrogenase, L-malate dehydrogenase and succinate dehydrogenase. This is in line with previous reports that inflammatory mediators and reactive oxygen or nitrogen species may affect mitochondrial functions in brain cells and in human chondrocytes [64,65,66,67]. The MeJA treatment did not modify the diminished L-malate dehydrogenase activity, but it prevented the diminished NADH dehydrogenase activity. Surprisingly the MeJA treatment further increased the mitochondrial succinate dehydrogenase, which was possibly increased in arthritic rats by the action of TNFα [63]. It is difficult to infer from the available data the consequences and significance of these actions of MeJA for the mitochondrial functions.

In the brain, arthritis did not modify the hexokinase activity, contrary to what happens in the liver where the activity of the analogous glucose phosphorylating enzyme (glucokinase) is substantially increased, leading to augmented hepatic glycolysis [9]. In the brain the treatment with MeJA resulted in an apparent increase in the hexokinase activity, a phenomenon that has already been described for cancer and brain cells [5]. This apparent increase when the hexokinase is assayed in the cytosolic fraction results from the detachment of the enzyme from the mitochondria. Although the phenomenon was only detected at high concentrations in the present work after 30 min incubation, it is worth mentioning that the phenomenon is time-dependent [5], which makes it a likely occurrence in animals exposed to low doses during several days. The enzyme isoforms normally attached to the mitochondrial membrane are types I and II. Type IV hexokinase (glucokinase), the isoform normally found in the liver cells, is not attached to the mitochondrial membrane [5]. Our results suggest that detachment of the hexokinase from the mitochondria in healthy and arthritic animals by MeJA was similar. Consequently, the phenomenon should in principle not be considered as a mechanism capable of having contributed to the antioxidant or prooxidant activities of MeJA. In cancer cells the detachment of the hexokinase from the mitochondria is considered one of the main reasons for its cytotoxicity because these cells depend heavily on the oxidation of glucose for survival and ATP for its phosphorylation is more readily available at the surface of the mitochondria [68,69]. Detachment of the hexokinase from the brain mitochondria by MeJA has not been considered neurotoxic however, probably because the brain cells are less dependent on glycolysis for survival [5], even though a limited impairment of glycolysis in the brain can be expected to occur.

The behavioral and cognitive changes induced by MeJA and reported by several authors require a final set of comments. MeJA improves the cognitive dysfunction associated with neuro-inflammation. It was reported that MeJA attenuates LPS-induced memory dysfunction via mechanisms involving inhibition of pro-inflammatory mediators and beta-amyloid generation in mice [27]. In other investigations the effects of MeJA were assessed in mice with memory impairment caused by scopolamine and unpredictable chronic mild stress (UCMS) [28,29]. In these studies, MeJA affected oxidative stress in the brain in a way that was similar to that found in the present study. In these studies, MeJA was intraperitoneally administered at doses of up to 100 mg/kg and the oxidative status improvement was associated with neuroprotection and reduced memory dysfunctions. This allows to hypothesize that the antioxidant and anti-inflammatory properties of MeJA may also improve the brain fatigue and cognitive function in patients with severe rheumatoid arthritis [12,24].

## 5. Conclusions

In conclusion, MeJA treatment decreased inflammation and oxidative stress in the brain of rats with adjuvant-induced arthritis. The improvement of oxidative stress occurred in consequence of reduced inflammation associated with increased antioxidant defenses. On the other hand, MeJA induced the hexokinase (HK) detachment from the brain mitochondria of both healthy and arthritic rats a phenomenon that can diminish glucose phosphorylation and metabolism in the brain. The effective doses of MeJA were relatively high, but the results of the present study make this compound a potential precursor for developing anti-rheumatic and anti-inflammatory drugs. Finally, future investigations may take into account the role of Nfr2 in the actions of MeJA and the perspective of modifications in the metabolism of glucose in the brain tissue.
